# Supplementary material for: Migration: A Neglected Potential Contribution of HCl-Oxidized Au(0)
Source: Molecules. 2023 Feb 7;28(4):1600. doi: 10.3390/molecules28041600 (PMC9964448; doi:10.3390/molecules28041600)
Supplement: Supplementary file 1 [file molecules-28-01600-s001.zip › molecules-2188070-supplementary.pdf]

molecules

Supplementary Materials

Migration: A Neglected Potential Contribution of HCl-Oxidized Au(0)

Zilong Zhang [1], Haifeng Zhang [1,*], Bolin Wang [1,2,*], Yuxue Yue [2] and Jia Zhao [2,*]

[1] School of Chemical Engineering, Northeast Electric Power University, Jilin 132012, China
[2] Industrial Catalysis Institute, Zhejiang University of Technology, Hangzhou 310014, China
* Correspondence: zhfeepu@163.com (H.Z.); bolinwang@neepu.edu.cn (B.W.); jiazhao@zjut.edu.cn (J.Z.)

Supplementary Experimental

Catalyst preparation

The catalysts were prepared by a classical wet impregnation method. The activated carbon (AW1101, KoTHmex, Taiwan Carbon Technology Co.) and $HAuCl_4 \cdot xH_2O$ (Alfa Aesar, 40 mg, assay 49%) aqueous solution were mixing in deionized water (H_2O, 18.25 MΩ cm^{-1}). The mixture was impregnation for 12 h and then dried at 120°C for 12 h, was designated Au/C, Au loading of 1.0 wt.%. Due to the nature of the catalyst preparation procedure used, wet impregnation, no filtration of the carbon or catalyst washing was carried out, and the metal loading should be considered as equal to the nominal amount of metal impregnated into the hosts.

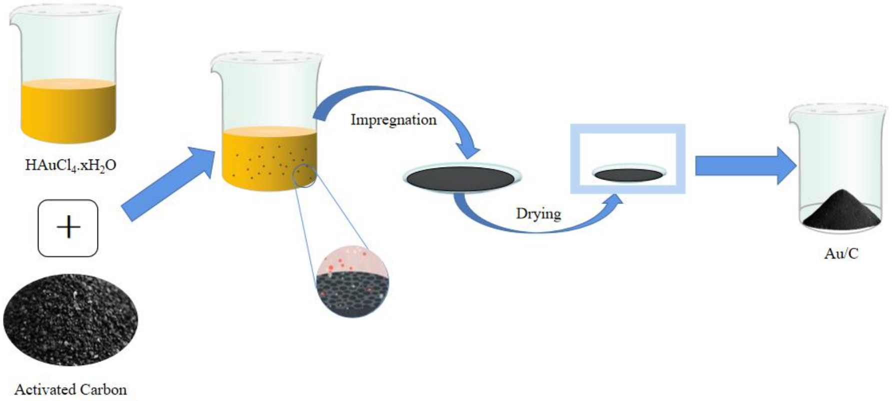

Figure S1. Preparation of the Au/C catalyst by wet impregnation.

Catalyst tests and characterization

Catalytic tests of HCl sequence pretreatment was carried out in a fixed bed glass reactor (i.d. 10 mm). First of all, a certain amount of catalyst was loaded in the reactor, followed by sweeping with nitrogen (N_2) gas at 393 K for at least 0.5 h to remove impurities like water and air, and then addition of HCl with a GHSV of 1000 h^{-1}, which was controlled via a mass flowmeter at a selected reaction temperature. The mixed gas obtained from the reactor outlet was washed by NaOH solution. The content of metals within the catalyst was analyzed by inductively coupled plasma atomic emission spectrometer (ICP-AES) with a PerkinElmer Elan DRC-e. X-ray photoelectron spectra (XPS) were acquired on a Kratos AXIS Ultra DLD spectrometer using monochromatic Al-Ka radiation, generated from an electron beam operated at 15 kV, and equipped with a hemispherical capacitor electron-energy analyser. The samples were analysed at the electron take-off angle of 45° and the pass energy of 46.95 eV. All binding energy values were referenced to the C 1s signal at 284.4 eV. Survey spectra of the Au/C catalyst with HCl sequence pretreatment were shown in Figure S1. The spectrometer was calibrated for the Au $4f_{7/2}$ signal at 84.0 ± 0.1 eV with a resolution step width of 0.2 eV. Temperature-programmed desorption (TPD) and Temperature pro-grammed reduction (TPR) analysis were measured on a thermal conductivity detector (TCD) and an Omnistar GSD320 mass spectrometer. The Au(III) amount was reported as a ratio of the Au(III) to the total Au amount using CuO as a standard. The released CO gasses was calibrated at the end of each measurement, calibrated using the known composition.

Computational details

All the calculations were implemented using the plane wave basis Vienna Ab-initio Simulation Package (VASP). The Perdew-Becke-Ernzerhof (PBE) parameterization of the Generalized Gradient Approximation (GGA) as the exchange-correlation functional was implemented. The kinetic energy cutoff for the plane wave basis sets was 500 eV to approximate the valence electron densities and the projector augmented wave (PAW) method to account for the core-valence interaction. The Brillouin-zone sampling was restricted to the Gamma point as we used a large computational cell. All investigated structures are presented in Fig. 2 and listed in supplementary Table S3. The simulations were carried out for a 32-atom carbon nanoribbons supercell. A 8.65×12.00×20.24 $Å^3$ hexagonal supercell of carbon nanoribbons was used for the x, y, and z directions. Carbon nanoribbons is periodic in the x direction, whereas a vacuum space of 10 Å is along the y and z directions. The force convergence criterion was set to 0.05 eV/Å in an optimized structure. The climbing image nudged elastic band (CI-NEB) method was used to find a minimum energy path along the reaction pathways. During the geometry relaxation, the shape and volume of the unit cell as well as the atomic positions in the unit cell of each configuration are fully optimized. The convergence threshold for self-consistent-field iteration is set to 10^{-5} eV, and the geometry optimization was iterated until all atomic forces became smaller than 0.02 eV/Å.

Table S1. Surface and bulk composition of Au/C catalyst.

Functional group	C [µmol g(Cat.)$^{-1}$]	C-HCl [µmol g(Cat.)$^{-1}$]
Carboxylic	36	59
Lactonic	4	16
Phenolic	232	241
Total acidic groups	272	316

Table S2. Bulk phenolic compositions of Au/C catalyst with different pretreatment from CO TPD-MS.

Sample	Phenolic group [µmol g(Cat.)$^{-1}$]
C	217
C-HCl	224
Au/C	155
Au/C-HCl 60 min	36

Table S3. Reaction energy for Au proposed migration process.

Step	Structure	Energy [eV]
	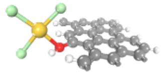	0.00
Eq. 1	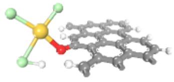	0.79
	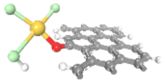	-0.28
	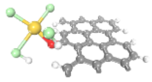	-0.52
Eq. 2	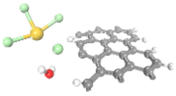	1.44
	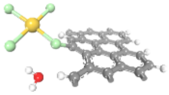	-1.00
	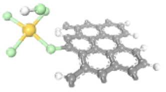	-0.54
Eq. 3	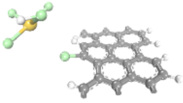	-0.09
	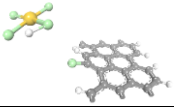	-0.66

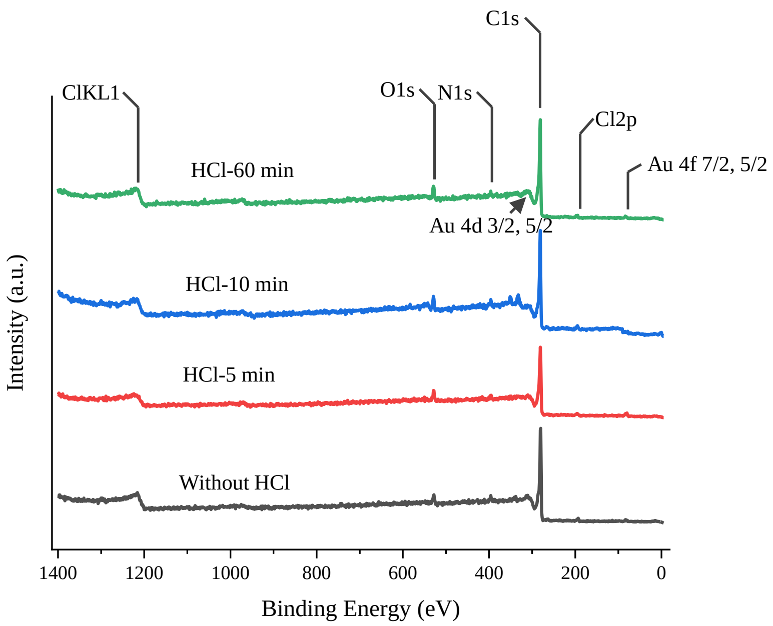

Figure S2. Survey spectra of the Au/C catalyst with HCl sequence pretreatment.

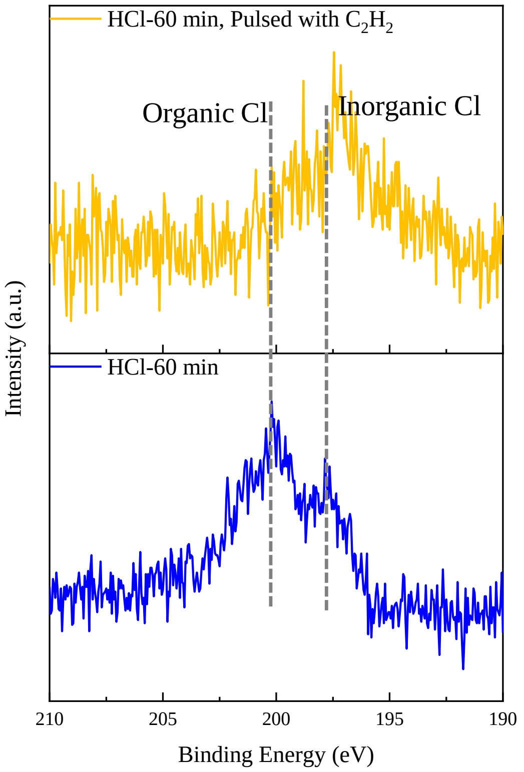

Figure S3. XPS spectrum of the Au/C catalyst with different treatment.

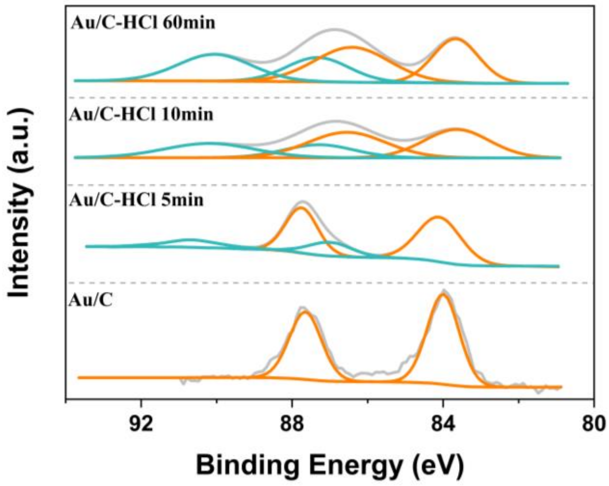

Figure S4. XPS spectrum and simulation for the Au/C catalyst with HCl sequence pretreatment (derived from Fig. 1d).

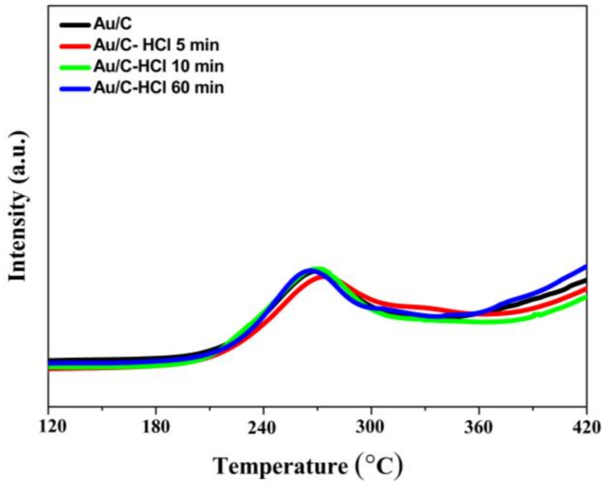

Figure S5. H_2-TPR profiles of Au/C with HCl sequence pretreatment. The reduction profile of Au/C appears as a single peak between 180 and 350 °C; this reduction peak has been assigned to the reduction of the Au(III) precursor formed during catalysts preparation (*Catal. Sci. Technol., 2013, 3, 128-134*). The Au(III) amount is reported as a ratio of the Au(III) to the total Au amount.

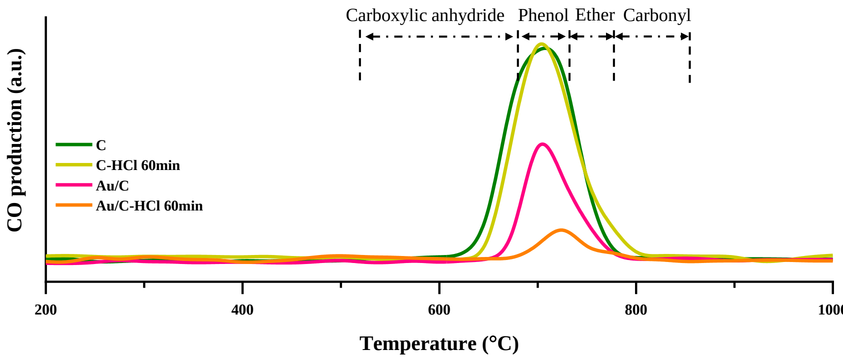

Figure S6. CO-TPD profiles of Au/C and reference C samples with HCl sequence pretreatment. The desorption CO profiles centered at ~700 °C region can be attributed to the phenolic groups (*J. Catal., 2018, 365, 153-162; Carbon, 2007, 45, 785-796; Carbon,1999, 37, 1379-1389*).

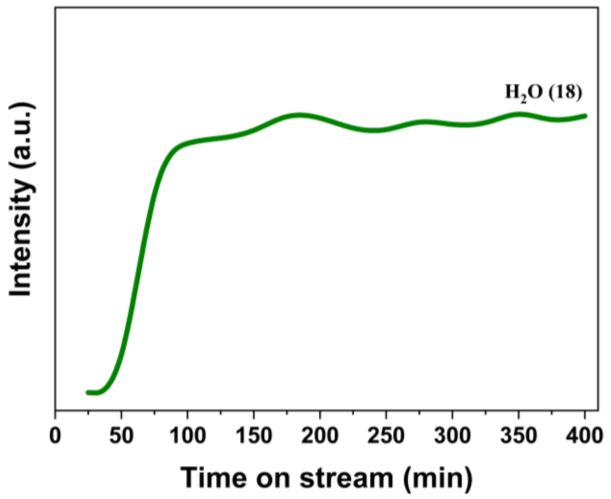

Figure S7. Detected desorption gas signals of Au/C with HCl pretreatment.

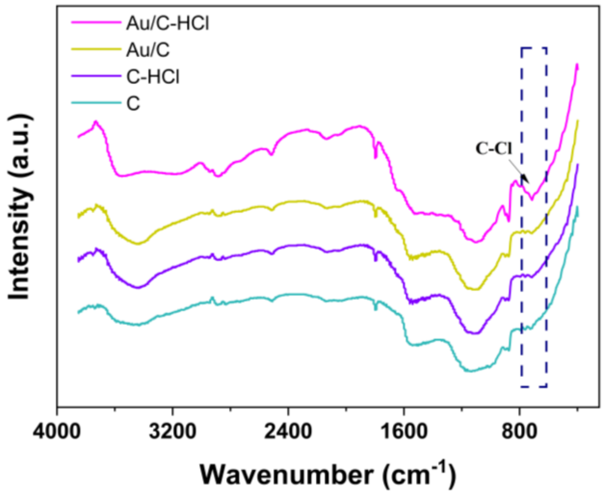

Figure S8. FTIR profiles of Au/C and reference C samples with HCl pretreatment.
